# Investigation of Natural Compounds for Therapeutic Potential in Streptozotocin-induced Diabetic Neuroinflammation and Neuropathic Pain

**DOI:** 10.3389/fphar.2022.1019033

**Published:** 2022-10-05

**Authors:** Muhammad Faheem, Arif-ullah Khan, Fawad Ali Shah, Shupeng Li

**Affiliations:** ^1^ Riphah Institute of Pharmaceutical Sciences, Riphah International University, Islamabad, Pakistan; ^2^ State Key Laboratory of Oncogenomics, School of Chemical Biology and Biotechnology, Shenzhen Graduate School, Peking University, Shenzhen, China

**Keywords:** natural compounds, molecular dynamic simulation, diabetic neuroinflammation, neuropathic pain, ELISA, PCR

## Abstract

Diabetic neuropathy (DN) is a serious microvascular complication of diabetes mellitus (DM) that impacts the nervous system. Several risk factors are involved in the progression and maintenance of DN-associated pain, such as higher expression of various inflammatory mediators, e.g., tumor necrotic factor-alpha (TNF-α), nuclear factor-kappa B (NF-κB), and cyclo-oxygenase-2 (COX-2). The present research explores the neuroprotective potential of natural isolates, including berbamine, bergapten, and carveol, on the DM-induced neuroinflammation and neurodegeneration that cause neuropathic pain. The study utilized computerized techniques, including computational analysis (a docking assay and a molecular dynamic simulation) before moving to *in vivo* protocols. Diabetic neuropathy was induced by intraperitonial injection (IP) of streptozotocin (65 mg/kg), and the animal subjects (rats) were kept for 4 weeks for the development of DN. Once diabetic neuropathy was confirmed, the subjects were treated with berbamine, bergapten, and carveol until the sixth week (i.e., 2 weeks of treatment). At the sixth week, the rats were sacrificed, and the sciatic nerve and spinal cord of each was collected for further molecular investigation. Docking and a molecular dynamic simulation (MDS) delivered the information that the natural compounds (berbamine, bergapten, and carveol) were interacting with the selected target protein (i.e., mitogen-activated protein kinase). After IP, it was found that berbamine, bergapten, and carveol had ameliorated mechanical allodynia and thermal hyperalgesia by the 28th day of the study (2 weeks after treatment) without affecting blood glucose levels. Berbamine, bergapten, and carveol markedly elevated the levels of glutathione (GSH) and glutathione s-transferase (GST), in both the sciatic nerve and spinal cord, and also reduced lipid peroxidase (LPO) and nitric oxide (NO). The abovementioned natural isolates reduced pathologic alterations provoked through DN, a finding confirmed through histopathological assays (hematoxylin and eosin staining and immuno-histochemical analysis). Treatment down regulated higher expressions of the inflammatory mediatorcyclooxygenase-2 (COX-2), tumor necrosis factor-α (TNF-α), and nuclear factor kappa B (NF-κB), as confirmed by ELISA and polymerase chain reaction (PCR). The outcomes of berbamine, bergapten, and carveol are compared with those of pregabalin as a positive control group. Compared to pregabalin, treatment with the aforementioned three natural compounds improved nociception and reduced hyperalgesic effects, and consequently reduced pain perception and inflammation. Our results suggest the mechanism for the neuro-protective impact of berbamine, bergapten, and carveol might possibly be arbitrated via COX-2, TNF-α, and NF-κB, and regulated by mitogen-activated protein kinase, ultimately ameliorating STZ-provoked, DM-induced neuroinflammation and neurodegeneration, and associated neuropathic pain.

## Introduction

**FIGURE 1:**
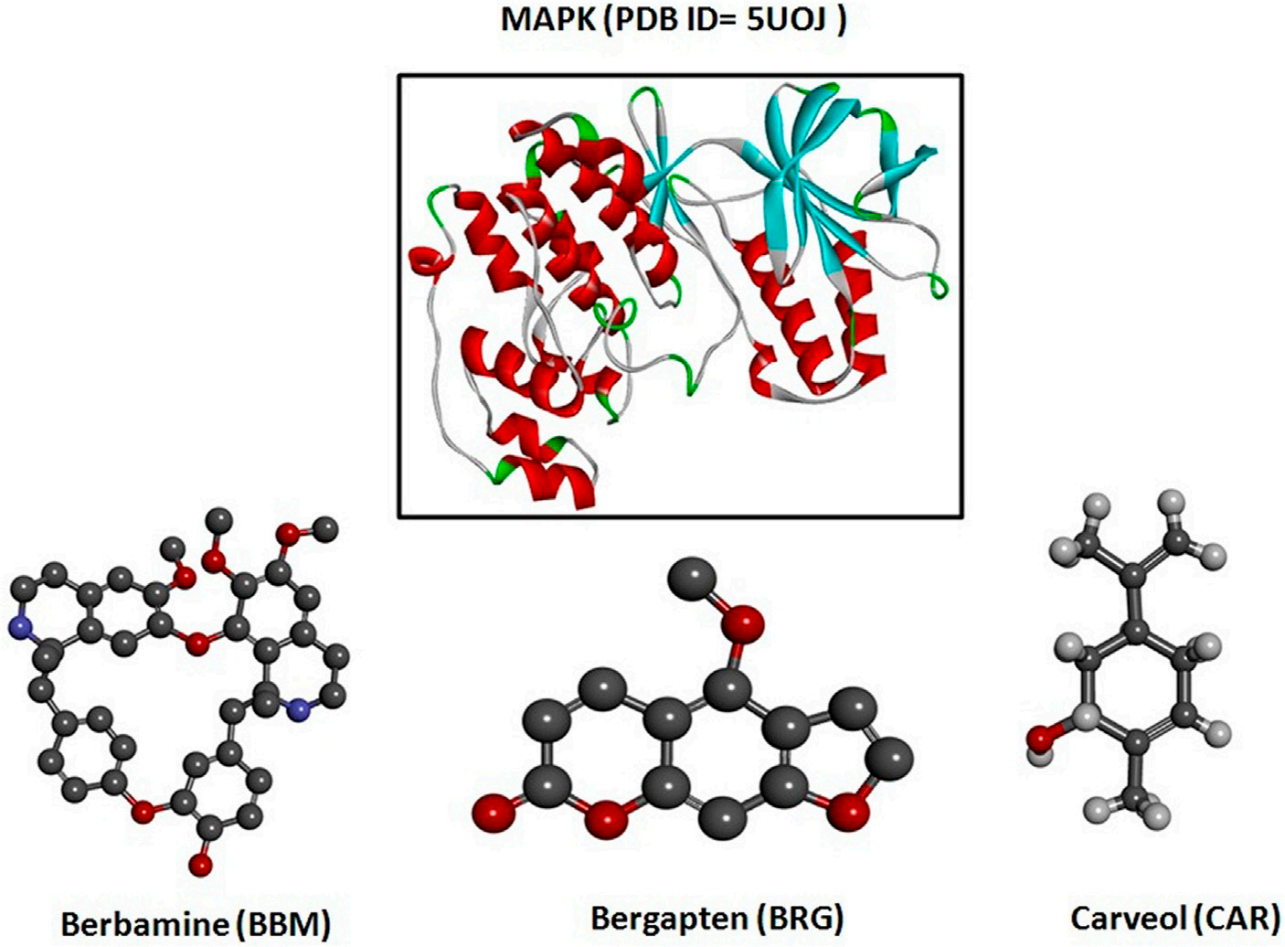
3D structures of berbamine, bergapten, and carveol and mitogen-activated protein kinase (MAPK, PDB I.D: 5UOJ) drawn through Chem Draw and Discovery Studio Visualizer client 2016.

Diabetes mellitus (DM) is a most serious metabolic syndrome, and the fifth leading cause of death worldwide, being characterized by persistent hyperglycemia (Go et al., 2015). According to the International Diabetes Federation (IDF), in 2021 Pakistan had a population of 33 million diabetics, which was 70% higher than in 2019. Furthermore, 26.9% of those affected with diabetes remain undiagnosed (IDF, 2021). DM can result in serious complications, influencing various organs, such as the nerves, kidneys, heart, blood vessels, and eyes (Wild et al., 2004). Diabetic neuropathy (DN) is a microvascular complication of persistent DM that affects the nerves and results in significant disease and death rates (Kallinikou et al., 2019). Estimates of the high prevalence of DN among those diagnosed with diabetes in Pakistan range from 36% to 68% (Ali et al., 2013). Distal symmetrical sensory neuropathy is the most widely known type of DN, or recognized polyneuropathy. Loss of motor and sensory nerves are attributes of DN. Ongoing hyperglycemia contributes to neurotic changes, such as tightening of the neuronal capillary, axonal condensing, demyelination of nerves, injury to nerve fibers, and neuronal damage (Sasaki et al., 2020). Studies of the etiology of DN have distinguished numerous bio-chemical mechanisms of nerve and neurovascular impairment, amongst which neuronal injury may be ascribed to elevated levels of oxidative stress (Suryavanshi et al., 2020). Inside the peripheral nervous system, both chronic and acute DM are sources of oxidative stress, and trigger the progression of DN (Heydari et al., 2014). Modifications in neuronal activity within the peripheral and central nervous systems, and the further stimulation of glial and immune cells, can lead to the pathogenesis of neuropathic pain. Peripheral nerve injury is accountable for the onset of numerous inflammatory arbitrators, as well as chemokines and cytokines significant for the development and preservation of neuropathic pain (Woolf and Ma, 2007). Hyperglycemia also stimulates the transcription nuclear factor (NF-κB), which in turn causes overexpression of specific gene sequences that control the regulation of numerous inflammatory cytokines, such as tumor necrosis factor-alpha (TNF-α), and cyclooxygenase-2 (COX-2) (Ahmed et al., 2016). The medical demonstrations of DN produce distinct neuropathic manifestations: impulsive pain like burning, electric shock or a penetrating sensation, thermal and mechanical hypersensitivity, or alternatively, lack of pain, but an accompanying lack of sensitivity (Boulton, 2012). The current research work was designed to assess the protective effect of berbamine, bergapten, and carveol on streptozotocin-induced DN in rats, targeting various proteins, such as mitogen-activated protein kinase (Figure 1) for *in silico* analysis, and nuclear factor kappa B (NF-κB), tumor necrosis factor-alpha (TNF-α), and cyclooxygenase-2 (COX-2) for *in vivo* investigations.

Hence, these compounds, i.e., berbamine (BBM), bergapten (BRG), and carveol (CAR), as shown in Figure 1, are explored for their potential therapeutic use in the neuropathic pain from STZ-induced diabetes. Natural compounds exhibit promising neuroprotective potential, as per recently published literature (Faheem et al., 2022a). Berbamine (BBM) is a bisbenzylisoquinoline alkaloid from *Berberis amurensis* Rupr. It possesses anti-cancerous and anti-inflammatory properties, multidrug resistance, and synergistic activities. Psoralen is a furocoumarin-derived natural chemical from the *Ammi majus* plant. Methoxsalen-based furocoumarin, including bergapten (5-methoxsalenpsoralen), has been researched in relation to cancer, vitiligo, and psoriasis. The caraway plant yields carveol (CAR), an essential oil ingredient cultivated globally. Caraway includes pinene, thujene, phellandrene, camphene, limonene, and carvone (Faheem et al., 2022b). The selection of these compounds is based on their anti-inflammatory potential. The compounds were administered and, according to the findings, therapy with these natural chemicals (BBM, BRG, and CAR) down regulated NF-κB, which resulted in an attenuation of the diabetic-induced neuropathic pain caused by STZ. The aforementioned natural substances are thought to work by inhibiting the activity of the transcription factor NF-κB, which in turn reduces the production of pro-inflammatory cytokines, such as COX-2 and TNF-α, which may be suggested as a mechanistic route. In addition, the treatment improved antioxidant enzymes (GSH and GST) and decreased LPO and iNOS, thus preventing oxidative stress and free radical generation, halting the progression of STZ-mediated DN, and hence attenuating neurodegeneration and neuroinflammation.

## Material and methods

### Chemicals

Chemicals used were streptozotocin (STZ) formaldehyde, ketamine, 5% dimethyl sulphoxide (DMSO), hydrogen peroxide (H2O2), di-hydro-dithiobis-nitrobenzoic acid (DTNB), lipid peroxidase (LPO), PBS tablets, normal saline,1-chlor-2,4-dinitrobenzene (CDNB), xylaxine, GST, proteinase K, reduced glutathione (GSH), streptozotocin (STZ), ethanol, trichloroacetic acid (TCA), liquid nitrogen, trizol, chloroform, isopropanol, 70% ethanol, RNase-free water, ELISA kits including NF-κb by E-lab sciences (Catalogue number: E-EL-R0674), TNF-α by E-lab sciences (Catalogue number: E-EL-R0019) and COX-2 by Nanjing pars Biochem (Catalogue number: PRS-30205Ra) and pregabalin. The chemicals were all purchased from Sigma Aldrich.

### Animals

Male Sprague-Dawley rats weighing 200–250 gm were utilized in the study. These rats were kept under a 12 h light–dark cycle in animal housing at the Riphah Institute of Pharmaceutical Sciences, Islamabad, with a precise environment (temperature: 25 ± 2°C and humidity: 55% ± 5%). The animals were allowed standard food and water *ad libitum*, and were divided into different groups for the experiments. All experimental work was executed in accordance with the guiding principles of the Institute of Laboratory Animal Resources, Commission on Life Sciences University, National Research Council (1996), and was accepted by the Ethical Committee of the Riphah Institute of Pharmaceutical Sciences (Ref. No. REC/RIPS/2019/28).

### Animal grouping and dosing

The rats were divided into 6 groups (*n* = 6 rats per group).

Group I (Non-Diabetic: ND) served as the saline group (NaCl 0.9%; 10 ml/kg) without STZ.

Group II (Diabetes Neuropathy: DN) served as a disease group (STZ; 65 mg/kg body weight).

Group III (DN + BBM) served as a treatment group (STZ; 65 mg/kg + BBM [1, 5, and 15 mg/kg]).

Group IV (DN + BRG) served as a treatment group (STZ; 65 mg/kg + BRG [25, 50, and 100 mg/kg]).

Group IV (DN + CAR) served as a treatment group (STZ; 65 mg/kg + CAR [5, 10, and 20 ml/kg]).

Group VI (DN + PBN) served as standard group (STZ; 65 mg/kg + pregabalin [PBN: 30 mg/kg]).

The dosing started 4 weeks after the induction of diabetes and continued until the sixth week. All behavioral investigations were performed after confirmation of STZ-induced diabetes mellitus associated DN on the 28th, 31st, 35th, 38th and 42nd days after induction. After six weeks the rats were sacrificed, and tissue samples of their sciatic nerves and spinal cords were collected for histopathological and molecular investigation.

### Docking studies

Molecular docking or computational analysis was carried out to study the different therapeutic effects of the test compound by focusing on good binding affinity at the target site. The docking studies of berbamine (BBM), bergapten (BRG), and carveol (CAR) were performed using Auto Dock Vina 4.0 version (Scripps Research, San Diego California) and PyRx (Scripps Research, San Diego California) software against designated targets involved in DN and inflammation, such as mitogen-activated protein kinase (MAPK PDB ID: 5UOJ). ChemSketch (http://www.acdlabs.com) was used to prepare the ligand, and afterwards, Discovery Studio Visualizer -2016 (DSV- 2016; Dasault System Biovia Corp) converted it into a three-dimensional structure saved in a protein data band (PDB) file. Target proteins were retrieved from the protein data bank in PDB format, and refined by the DSV 16. The docking was performed through selection of amino acid residue from the active site and not against entire proteins; and binding affinity value was articulated in kcal/mol. PyRx software was used to obtained the best pose for the ligand receptor complex. The schematically depicted hydrogen bonding, hydrophobic bonds, and amino-acid residues participating in H-bonding of the best docked pose for the ligand protein-complex was achieved through post docking analysis (Malghani et al., 2020).

### Molecular dynamic simulation

Molecular dynamics simulations of three complexes, i.e., BBM-MAPK, BRG- MAPK, and CAR-MAPK, were performed using the Desmond software package (Ivanova et al., 2018). Complexes were first exposed to an orthorhombic box with their respective number of water molecules for complexes by a simple point charge water model, with optimized potential for liquid simulations. After exposure to an isotonic environment through the addition of NaCl, the system was made electrically stable by adding counter ions, a Nose–Hoover thermostat maintaining a 300-k temperature, and pressure of 1.01325 bars by Martyna–Tobias–Klien barostate with 100 ns as the time of the simulations. Determination of electrostatic interactions was performed using the Mesh Ewald method (Faheem et al., 2021).

### Induction and evaluation of diabetes and diabetic neuropathy in rats

After overnight fasting, the rats were subjected to a single intraperitoneal (IP) injection of streptozotocin (STZ; 65 mg/kg) solution for induction of DM. Blood samples were collected from the rats’ tail veins 48 h after STZ administration and assessed for plasma glucose levels (PGLs) with an Accu-Check Performa gluco-meter. The rats with PGLs above 400 mg/dl were taken into consideration (Ostovar et al., 2020). After 28 days, treatment was initiated and was continued till 14th day. The animals were subjected for behavioral assays (mechanical and thermal hyperalgesia) and then molecular investigation (Yadav et al., 2014).

### Plasma glucose levels assessment

In the present study, the PGLs of the rats were taken into consideration as metabolic parameters. The PGLs of the rats in each group were estimated after 48 h, and on the 14th day and the 28th day after STZ administration (Selagzi et al., 2008).

### Mechanical allodynia

Von Frey filaments were used to assess the mechanical allodynia on the 14th day of treatment (Iqbal et al., 2020). After habituation, the rats were placed in a cage with a mesh floor, and the Von Frey filaments were applied from below the mesh floor on the mid planter surface of each hind paw. Force was applied with lower pressure increasing to higher pressure perpendicularly on hind paws following filament bends of 2–3 s. Abrupt withdrawal responses of paws were observed.

### Thermal hyperalgesia

After habituation, thermal hyperalgesia was evaluated by placing the hind paws of the rats on a hot-plate, adjusted to a constant temp (55 ± 0.5°C) on the 28th day for confirmation of DN (Suryavanshi et al., 2021). The cutoff time was 20 s to avoid paw tissue damage (Khairnar et al., 2020). Increased sensitivity to thermal stimulus was estimated through this hot-plate test.

### Antioxidant assays

Investigation of oxidative stress markers in the sciatic nerves and spinal cords of the rats was significant in discerning the damage caused by STZ-induced DN. A tissue sample from both the spinal cord and sciatic nerve of each rat was homogenized in a phosphate buffer containing phenylmethylsulfonyl fluoride (PMSF) as protease inhibitor at 4°C. Centrifugation of the sample homogenate of both the sciatic nerve and spinal cord was performed at 3,000× *g* for 10 min and the supernatant separated and used for further investigation (Imran et al., 2020).

### Glutathione-S-transferase (GST) activity

1-Chloro-2,4-dinitrobenzol (CDNB) was used as a substrate to determine GST activity in homogenized tissue samples, as demonstrated previously, with a slight amendment (Shah et al., 2018). Each well was filled with 20 µl of the collected supernatant, a freshly prepared solution of 20 µl of 1 mM CDNB, 100 µl of buffer solution, and 15 µl of 5 mM reduced glutathione. An ELISA micro plate reader with a 412-nm wavelength was used to measure the absorbance of GST at room temperature.

### Reduced glutathione (GSH) activity

The decreased levels of GSH were determined by a method employed previously, but with a slight modification (Shah et al., 2018). Each well was filled with 10 µl supernatant mixed with 75 μl of 0.6 mM 5,5-dithio-bis (2- nitrobenzoic acid) (DTNB) in 0.2 M sodium-phosphate buffer with pH of 8, and after that 100 µl of phosphate buffer was added to each well. The 0.2 M phosphate buffer and 0.6 M DTNB solution was used as control. The absorbance of all samples was measured using the ELISA micro plate reader at a wavelength of 340 nm at room temperature. The purpose was to determine the real absorbance from the tissue sample, subtracting the absorbance of the control, which then implies the GSH level.

### LPO assay

Lipid peroxidation was performed employing the previously used protocol with a slight modification to estimate the thiobarbituric acid (TBA) reactive substances from tissue samples of the sciatic nerves and spinal cords of rats by colorimetric method (Shah et al., 2018). The tissue samples from each rat were homogenized separately. Centrifugation of sample homogenates was carried out at 3,000× *g* for 10 min and the LPO assay supernatant was collected. In this assay, 30 µl of the collected supernatant was mixed with 10 µl ferric chloride, 20 µl ascorbic acid and 36 µlk + buffer. The above mixture was then incubated in a water bath at 37°C for 1 h. To halt the reaction, 65 µl 0.8% TBA and 65 µl of trichloroacetic acid (TCA) were added, and this mixture was incubated at 100°C in a water bath for 20 min. The tubes containing samples were placed into ice-cold water and then centrifuged for 10 min at 2,500× *g*. The supernatant was then measured at a wavelength of 540 nm using a plate reader to estimate the absorbance of TBARS.

### Nitric oxide assay

After homogenization in a phosphate buffer, containing phenyl methyl sulfonyl fluoride as a protease inhibitor, the sample (of sciatic nerve and spinal cord) was centrifuged at 4,000×g for 10 min at 4°C, and the supernatant was collected and processed for determination of nitric oxide (NO) (Kumar et al., 2010).

### Histological examination

For histopathological examination and extraction of the sciatic nerve, the rats were sacrificed the sixth week after DM induction. The entire sciatic nerve of each rat was kept in a solution of 4% formaldehyde for fixing. Subsequently, the tissues were sectioned at 4 um coronal sections, cut using a rotary microtome, and fixed in a 4% formaldehyde solution. Later the fixed sciatic nerve sections were paraffinized for the following morphological analysis.

### Immuno-histochemical investigation

An immuno-histochemical (IHC) investigation was performed according to a previously well-defined procedure with slight modifications (Faheem et al., 2020). The tissue (spinal cord) was fixed in paraffin on slides, deparaffinized with xylene 100%, and rehydrated by washing with graded dilutions of ethanol (100%, 90%, 80%, and 70%). Slides were processed via enzymatic procedure for antigen retrieval with the addition of proteinase K. PBS was used to wash the tissue sample slides for 5 min and this was repeated three times. To extinguish endogenous peroxidase action, the slides were submerged in 3% hydrogen-peroxide for 10 min at room temperature. PBS was used to wash the slides again, and then blocking serum, such as 5% normal goat serum (NGS), was applied to every slide containing test tissues at room temperature and they were kept for incubation for 2 h. Subsequent slides were incubated with the primary antibodies, which included anti-NF-κb, anti-TNF-α, and anti-COX-2, and kept for overnight incubation at 4°C. The next day, 0.1 M PBS was utilized to wash the slides and repeated 2 times, before they were again incubated with biotinylated secondary antibodies for 1.5 hours at room temperature. Again, PBS was used to wash the slides, and a humidifier box was then used for incubation, after application of avidin–biotin complex (ABC) reagents to the slides for 60 min. The solution of 0.1% diaminobenzidine peroxidase (DAB) was used for staining the slides after washing with 0.1 M PBS. The sample slides were washed with deionized water then dehydration was carried out with graded conc. (70%, 80%, 90%, and 100%) of ethanol. Slides were dried in the open air, cleared with xylene, and mounting media was used for fixing the cover slips. IHC images of the slides were taken with a light-microscope in TIF format and then ImageJ software was used for further quantification.

### ELISA analysis

Commercially available ELISA kits were utilized to estimate expression of inflammatory markers, including NF-κb, COX-2, and TNF-α, as per the manufacturer’s instructions. Silent Crusher-M (Heidolph-Germany) was used to homogenize approximately 50 mg tissue samples of the sciatic nerve stored at-80°C, using 0.1 M PBS containing protease inhibitor as PMSF (phenylmethylsulfonyl-fluoride). The centrifugation for the subsequent homogenate was performed for 20 min at 3000 RPM at 4°C and then the supernatant carefully separated from the top-evading pallet at the bottom. Using a bi-cinchoninic acid kit (BCA), the protein conc. was measured for every group. The 96-well plates containing the supernatant of sciatic nerve samples were processed with targeted antibodies. The ELISA micro plate reader was used to estimate expression of inflammatory markers, which are TNF-α and COX-2. The readings were taken 3 times by repeating the procedure. The resultant values of the inflammatory markers were expressed in picograms per milliliter to the overall protein content (Faheem et al., 2022a).

### Real-time polymerase chain reaction

Following the instructions of the manufacturer, 200 mg of frozen tissue from the spinal cord was weighed and finely crushed in a prechilled pestle and mortar with liquid nitrogen. Homogenized tissue was kept at room temperature. Then, 1 ml of Trizol was added to the homogenate and transferred to 1.5 ml microfuge tubes. Tubes were gently inverted 4–5 times. The homogenate was incubated at room temperature for 5 min, 400 µl of chloroform was added and incubated at RT for 3 min, and then centrifuged at 12,000 rpm for 10 min at 4°C for phase separation. The aqueous upper layer was transferred to a new 1.5-ml tube placed on ice, and isopropanol was added in equal ratio. Tubes were incubated on ice (−20°C) for 10 min in a horizontal position to precipitate down RNA. Samples were centrifuged at 4°C and 12,000 rpm for 10 min. The pellets were washed twice with 1 ml of 70% ethanol at 7,500 rpm for 5 min at 4°C, and air dried completely. Then, 40 µl of RNase-free water or DEPC was added. RNA can be stored at −80°C until downstream application. cDNA was amplified by real-time PCR, using the same amount of cDNA, and employing a thermocycler as the method of execution. The expression of the mRNA was standardized according to the levels of expression of GADPH. The 2^ΔΔ-CT^ technique was used for real-time quantitative PCR, which allows for the determination of the relative gene expression (Imran et al., 2021). The forward and reverse primer sequences of rat GAPDH and TNF-α are shown as follows.

**Table:** 

Primer	Tm	Sequence (5′-3′)
Rat_GAPDH_F	62.5°C	TCTTCCAGGAGCGAGATCCC
Rat_GAPDH_R	62.5°C	TTCAGGTGAGCCCCAGCCTT
Rat_TNF_α_F	56.1°C	CTTCAAGGGACAAGGCTG
Rat_TNF_α_R	56.1°C	GAGGCTGACTTTCTCCTG

### Statistical analysis

The outcomes from the current study have been stated as mean ± standard error of the mean (SEM). The results of the behavioral studies were investigated through one-way ANOVA accompanied by *post hoc* Tukey test. GraphPad Prism 8.0 software (United States) was used to evaluate graphical data. The statistically significant difference was considered for values of *p* < 0.05 (symbols; *or#). Morphological statistics of the sciatic nerve were examined by ImageJ software (United States, version 1.46). The significant difference of the DN group compared to the saline group is denoted by symbol “#,” whereas the significant difference of the treatment groups compared to the DN group is denoted by symbol “*.”

## Results

### Molecular docking

**FIGURE 2:**
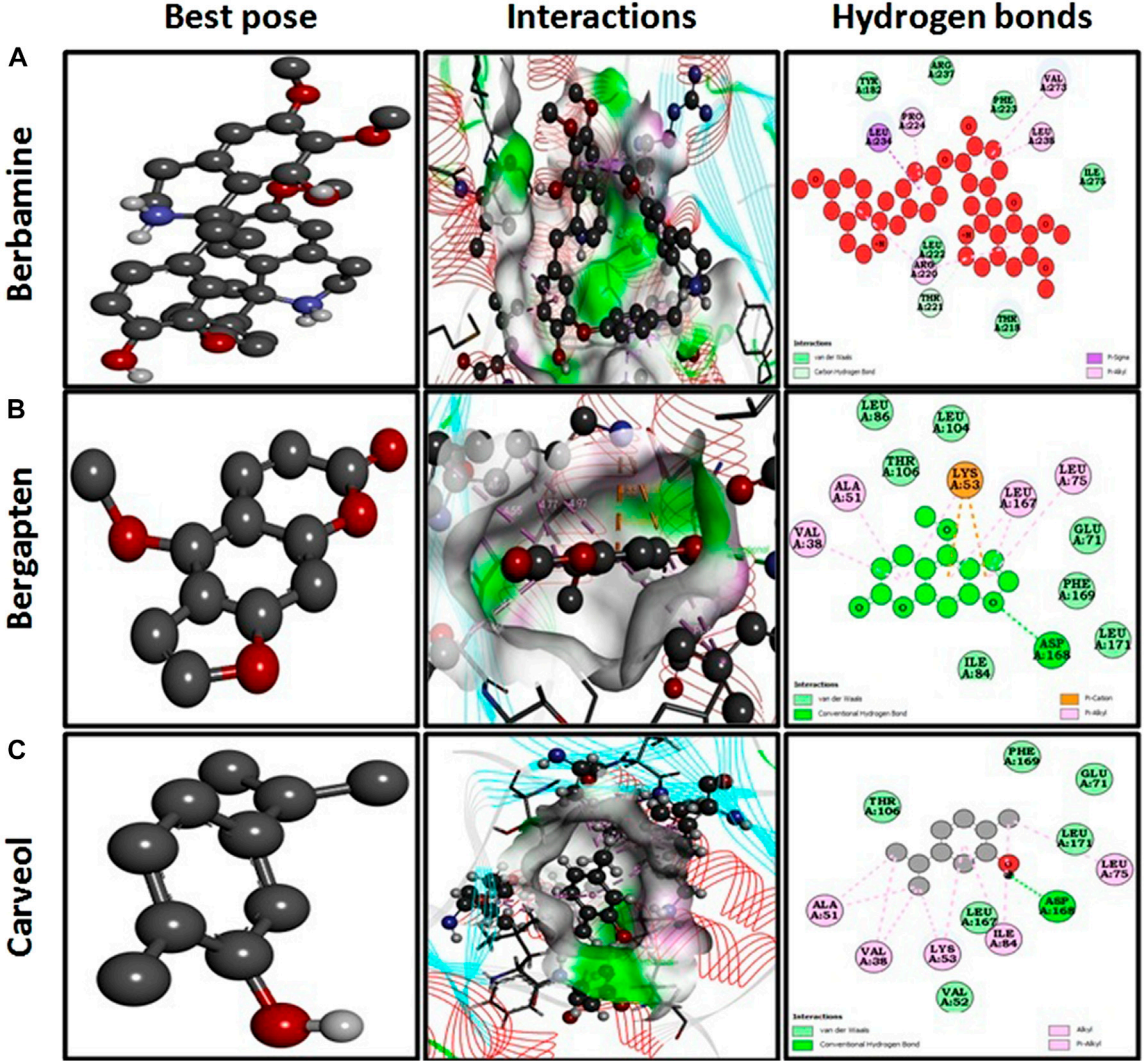
Represents best pose, interactions, and hydrogen bonds of berbamine **(A)**, bergapten **(B)** and carveol **(C)** and pregabalin against mitogenactivated protein kinase (MAPK, PDB I.D: 5UOJ) obtained through DSV 2016.

Docking investigations of the ligands berbamine, bergapten, and carveol were performed against proteins targets MAPK. Virtual results showed that berbamine, bergapten, and carveol possess binding affinity of 6.9, 6.5, and 5.8 kcal/mol against MAPK. Bergapten and carveol also formed one hydrogen bond against MAPK at ASP 168, as shown in Figures 2A–C.

### Molecular dynamic simulation

**FIGURE 3:**
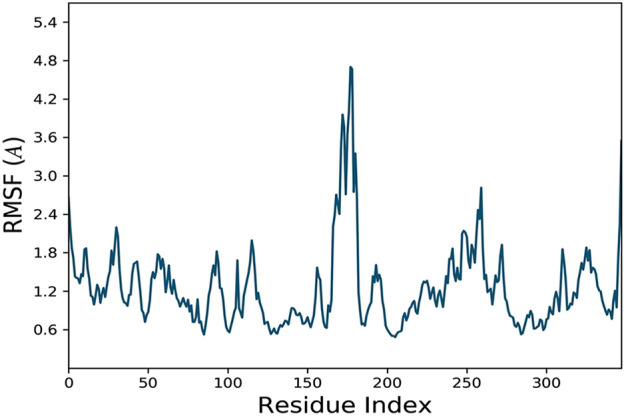
Represents root mean square fluctuation (RMSF) of mitogen-activated protein kinase (MAPK, PDB I.D: 5UOJ) obtained through Desmond software package.

**FIGURE 4:**
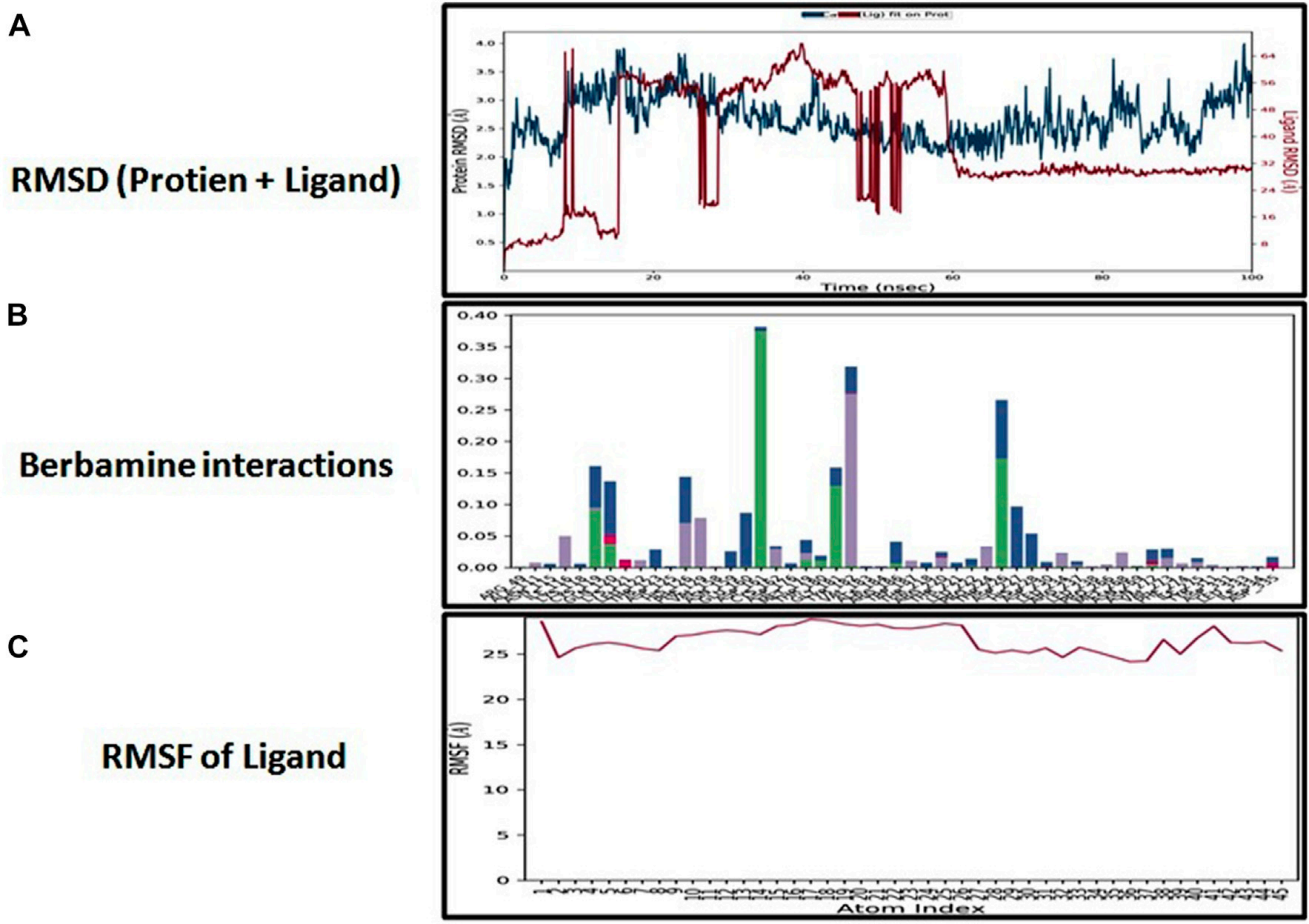
Represents root mean square deviation (RMSD) of mitogen-activated protein kinase (MAPK, PDB I.D: 5UOJ) and berbamine **(A)**, interactions (hydrogen bonds, hydrophobic interaction ionic and water bridges) of berbamine **(B)**, and root mean square fluctuation (RMSF) of berbamine **(C)** obtained through Desmond software package.

**FIGURE 5:**
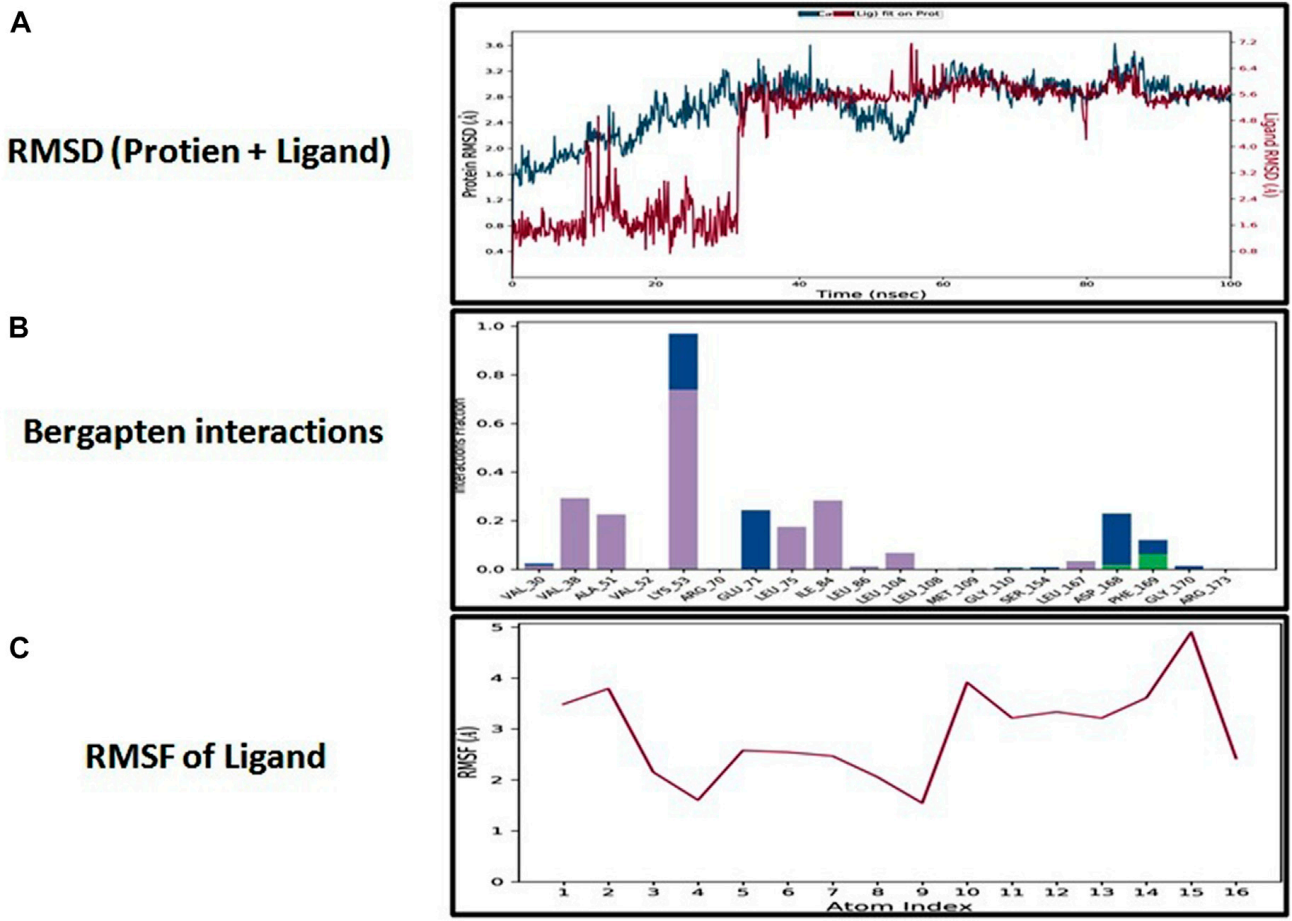
Represents root mean square deviation (RMSD) of mitogen-activated protein kinase (MAPK, PDB I.D: 5UOJ) and bergapten **(A)**, interactions (hydrogen bonds, hydrophobic interaction ionic and water bridges) of bergapten **(B)**, and root mean square fluctuation (RMSF) of bergapten **(C),** obtained through Desmond software package.

**FIGURE 6:**
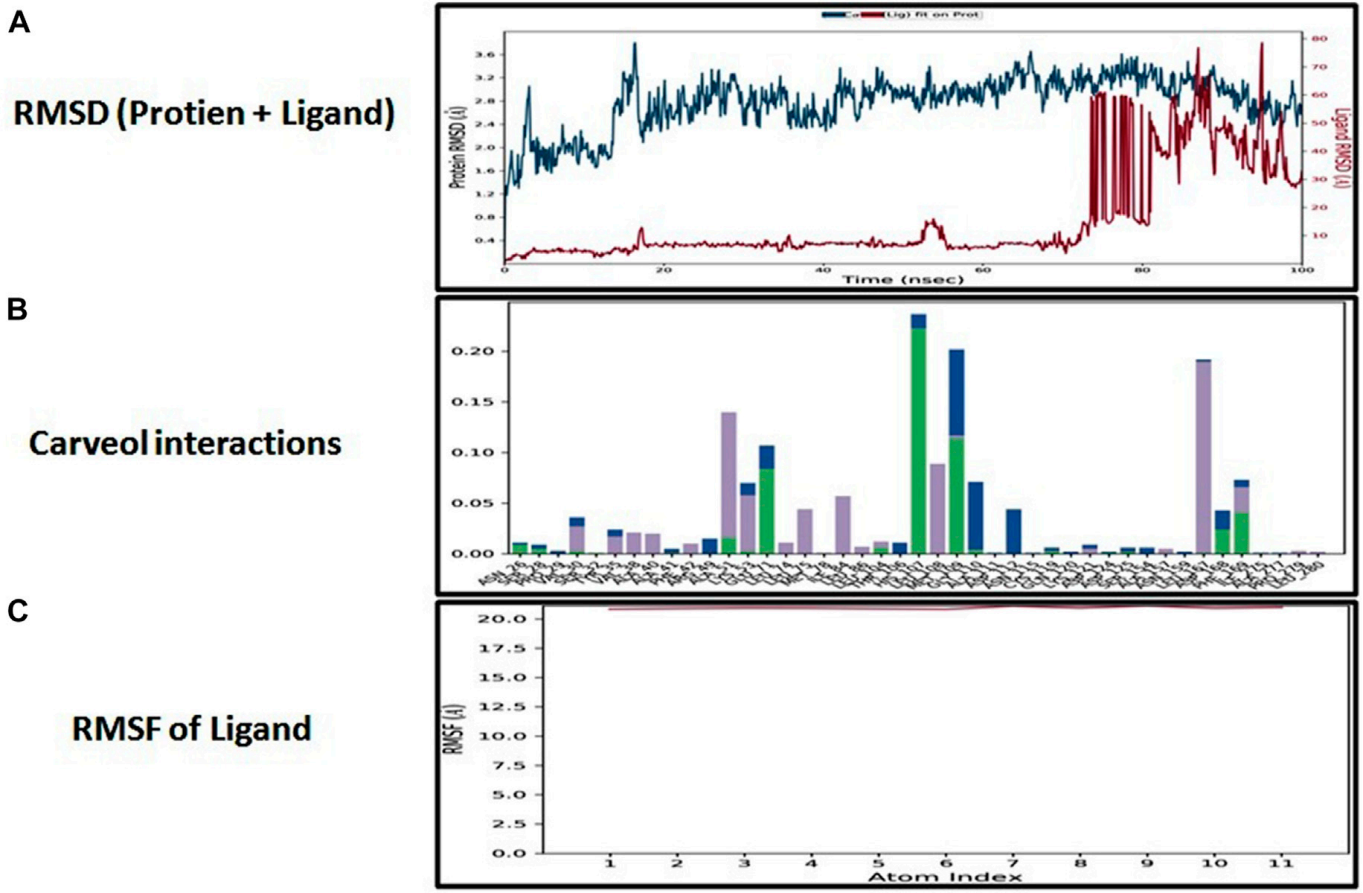
Represents root mean square deviation (RMSD) of mitogen-activated protein kinase (MAPK, PDB I.D: 5UOJ) and carveol **(A)**, interactions (hydrogen bonds, hydrophobic interaction ionic and water bridges) of carveol **(B)**, and root mean square fluctuation (RMSF) of carveol **(C)**, obtained through Desmond software package.

The docking analysis was further elaborated through molecular dynamic (MD) simulation, utilizing a Desmond software package. MD simulations of complexes of BBM-MAPK, BRG-MAPK, and CAR-MAPK were performed for 100 ns under a physiological environment. The results of the Desmond software package were received in the form of root mean square fluctuations (RMSF) of protein, as shown in Figure 3. The result for BBM in the form of the RMSD, hydrogen bonds, and the RMSF of the ligand are displayed in Figure 4. The BBM is interacting with the MAPK along the trajectory time of the simulation (100 ns) multiple times, as shown by Figure 4A. The BBM formed a hydrogen bond with multiple amino acids, including CYS119, GLY120, ASP161, MET179, THR 180, GLY181, THR226, and ASN276, as shown in Figure 4B. The result show a high RMSF, indicating reversible binding and less stability of BBM with the MAPK on the aforementioned environmental parameter, as shown in Figure 4C. The result for BRG in the form of the RMSD, hydrogen bonds and the RMSF of the ligand are displayed in Figure 5. The BRG is engaged with the target protein for more than 75% of the entire simulation duration, as shown in Figure 5A, providing stable hydrogen bonds at ASP168 and PHE169, as shown in Figure 5B. The result show reversible binding of BRG against the MAPK, but unlike BBM, BRG provides stable interactions, as reflected in the range of the RMSF values, as shown in Figure 5C, and in its hydrogen bonding. The result for CAR in the form of the RMSD, hydrogen bonds, and the RMSF of the ligand are displayed in Figure 6. CAR shows 35% engagement with the target protein, as shown in Figure 6A, proving hydrogen ASN26, SER28, VAL51, ALA107, GLU109, MET110, ASP168, and PHE189, as shown in Figure 6B. The result shows reversible binding of CAR against the MAPK, indicating unstable interactions, as reflected by the high RMSF value shown in Figure 6C.

### Plasma glucose level

The PGL was obtained at 48 h, and on the 14th day and the 28th day after STZ administration, and it was observed that the PGL was significantly high compared to the non-diabetic group, as shown in Table 1.

**TABLE 1:** Effect of berbamine (BBM), bergapten (BRG), carveol (CAR), and pregabalin (PBN) on blood glucose level (mg/dl) of rats at 48 h, 14th day, and 28th day after streptozotocin (STZ)-induced diabetic neuropathy. One-way ANOVA followed by *post hoc* Tukey test. ^###^ *p* < 0.001 vs. sham.

Plasma glucose level after 48 h					
ND (10 ml/kg)	DM (65 mg/kg)	BBM (1 mg/kg)	BBM (5 mg/kg)	BBM (15 mg/kg)	PBN (30 mg/kg)
150.3 ± 3.1	450.1 ± 2.5 ###	480.3 ± 2.2	428.4 ± 2.2	433.3 ± 2.1	423.9 ± 1.8
ND (10 ml/kg)	DM (65 mg/kg)	BRG (25 mg/kg)	BRG (50 mg/kg)	BRG (100 mg/kg)	PBN (30 mg/kg)
150.3 ± 3.1	450.1 ± 2.5 ###	490.5 ± 1.6	440.2 ± 2.8	490.8 ± 1.5	423.9 ± 1.8
ND (10 ml/kg)	DM (65 mg/kg)	CAR (5 ml/kg)	CAR (10 ml/kg)	CAR (20 ml/kg)	PBN (30 mg/kg)
150.3 ± 3.1	450.1 ± 2.5 ###	410.2 ± 3.1	480.1 ± 1.0	455.7 ± 3.2	423.9 ± 1.8
**Plasma glucose level after 14 days**					
ND (10 ml/kg)	DM (65 mg/kg)	BBM (1 mg/kg)	BBM (5 mg/kg)	BBM (15 mg/kg)	PBN (30 mg/kg)
153.2 ± 2.1	455.7 ± 4.0 ###	458.6 ± 1.8	468.4 ± 3.2	453.3 ± 2.1	442.3 ± 2.8
ND (10 ml/kg)	DM (65 mg/kg)	BRG (25 mg/kg)	BRG (50 mg/kg)	BRG (100 mg/kg)	PBN (30 mg/kg)
153.2 ± 2.1	455.7 ± 4.0 ###	472.2 ± 2.4	470.2 ± 4.8	420.8 ± 1.5	442.3 ± 2.8
ND (10 ml/kg)	DM (65 mg/kg)	CAR (5 ml/kg)	CAR (10 ml/kg)	CAR (20 ml/kg)	PBN (30 mg/kg)
153.2 ± 2.1	455.7 ± 4.0 ###	425.8 ± 2.8	445.1 ± 2.0	465.7 ± 3.2	442.3 ± 2.8
**Plasma glucose level after 28 days**					
ND (10 ml/kg)	DM (65 mg/kg)	BBM (1 mg/kg)	BBM (5 mg/kg)	BBM (15 mg/kg)	PBN (30 mg/kg)
153.2 ± 2.1	446.6 ± 4.1 ###	465.4 ± 2.9	478.4 ± 1.2	420.3 ± 4.1	435.8 ± 3.5
ND (10 ml/kg)	DM (65 mg/kg)	BRG (25 mg/kg)	BRG (50 mg/kg)	BRG (100 mg/kg)	PBN (30 mg/kg)
153.2 ± 2.1	446.6 ± 4.1 ###	488.8 ± 4.3	480.2 ± 1.8	472.8 ± 1.8	435.8 ± 3.5
ND (10 ml/kg)	DM (65 mg/kg)	CAR (5 ml/kg)	CAR (10 ml/kg)	CAR (20 ml/kg)	PBN (30 mg/kg)
153.2 ± 2.1	446.6 ± 4.1 ###	460.1 ± 2.3	462.1 ± 1.5	485.7 ± 3.2	435.8 ± 3.5

### Effect of paw withdrawal threshold

The effects of berbamine (BBM), bergapten (BRG), carveol (CAR), and pregabalin (PBN) on mechanical allodynia on the 28th day of streptozotocin (STZ)-induced diabetic neuropathic pain are shown in Table 2. The treatment with natural substances led to a considerable improvement in mechanical performance. In a nutshell, BBM enhanced the paw withdrawal threshold (PWT) compared to the STZ-induced neuropathic pain group, at doses of 5 and 15 mg/kg on day 28. When compared to STZ-induced neuropathic pain, BRG substantially increased the PWT at doses of 50 and 100 mg/kg on day 28. In comparison to STZ-induced neuropathic pain, the PWT was considerably improved by CAR on day 28 at both 10 and 20 ml/kg.

**TABLE 2:** Effect of berbamine (BBM), bergapten (BRG), carveol (CAR), and pregabalin (PBN) on 14th day on mechanical allodynia and thermal hyperalgesia in streptozotocin (STZ)-induced diabetic neuropathy.

Mechanical allodynia (after 14 days of treatment)					
ND (10 ml/kg)	DM(65 mg/kg)	BBM (1 mg/kg)	BBM (5 mg/kg)	BBM (15 mg/kg)	PBN (30 mg/kg)
38.4. ± 1.8	8.2 ± 2.5##	8.6 ± 2.0	15.4 ± 1.2*	31.4 ± 1.5**	28.8 ± 3.2**
ND (10 ml/kg)	DM(65 mg/kg)	BRG (25 mg/kg)	BRG (50 mg/kg)	BRG (100 mg/kg)	PBN (30 mg/kg)
38.4 ± 1.8	8.2 ± 2.5##	6.6 ± 1.5	14.2 ± 2.2*	29.30 ± 1.1**	28.8 ± 3.2**
ND (10 ml/kg)	DM(65 mg/kg)	CAR (5 ml/kg)	CAR (10 ml/kg)	CAR (20 ml/kg)	PBN (30 mg/kg)
38.4 ± 1.8	8.2 ± 2.5##	7.9 ± 2.4	16.8 ± 1.8*	30.9 ± 3.1**	28.8 ± 3.2**
**Thermal hyperalgesia (after 14 days of treatment)**					
ND (10 ml/kg)	DM(65 mg/kg)	BBM (1 mg/kg)	BBM (5 mg/kg)	BBM (15 mg/kg)	PBN (30 mg/kg)
15.5 ± 1.5	3.8 ± 2.5###	4.0 ± 2.2	7.8 ± 1.6*	12.3 ± 1.1**	10.2 ± 1.0**
ND (10 ml/kg)	DM(65 mg/kg)	BRG (25 mg/kg)	BRG (50 mg/kg)	BRG (100 mg/kg)	PBN (30 mg/kg)
15.5 ± 1.5	3.8 ± 2.5###	4.6 ± 1.6	6.9 ± 1.2*	11.0 ± 1.8**	10.2 ± 1.0**
ND (10 ml/kg)	DM(65 mg/kg)	CAR (5 ml/kg)	CAR (10 ml/kg)	CAR (20 ml/kg)	PBN (30 mg/kg)
15.5 ± 1.5	3.8 ± 2.5###	4.3 ± 3.1	8.8 ± 2.2*	11.8 ± 1.2**	10.2 ± 1.0**

The data were expressed as mean ± SEM (*n* = 6). One-way ANOVA followed by *post hoc* Tukey test. ^##^ *p* < 0.01, ^###^ *p* < 0.001 vs. sham and ^*^ *p* < 0.05, ^**^ *p* < 0.01 vs. STZ-induced diabetic neuropathic pain.

### Effect on thermal hyperalgesia

Table 2 displays the effects that berbamine (BBM), bergapten (BRG), carveol (CAR), and pregabalin (PBN) had on thermal hyperalgesia (TH) on the 28th day of streptozotocin (STZ)-induced diabetic neuropathic pain. The therapy with natural chemicals resulted in a substantial rise in TH. When compared with STZ-induced neuropathic pain, BBM increased paw TH at 5 and 15 mg/kg on day 28. BRG substantially increased TH when compared to STZ-induced neuropathic pain at doses of 50 and 100 mg/kg on day 28. When compared to STZ-induced neuropathic pain, the therapeutic effect of CAR at 10 and 20 ml/kg on day 28 was substantially more beneficial.

### Effect on oxidative stress markers

Table 3 displays the effects of berbamine (BBM), bergapten (BRG), carveol (CAR), and pregabalin (PBN) on streptozotocin (STZ)-induced DN, as well as the expression of glutathione (GSH), glutathione s-transferase (GST), inducible nitric oxide (iNOS), and lipid peroxidase (LPO). Results show that BBM, BRG, and CAR enhanced the level of protective markers (GSH and GST), while decreasing the harmful oxidative markers (LPO and iNOS). This may be the reason why therapy was able to reduce the neuroinflammation and neuropathic pain associated with STZ-induced neuropathic pain.

**TABLE 3:** Represents the effect of berbamine (BBM), bergapten (BRG), carveol (CAR), and pregabalin (PBN) on streptozotocin (STZ)-induced diabetic neuropathy and expression of glutathione (GSH), glutathione s-transferase (GST), inducible nitric oxide (iNOS), and lipid peroxidase (LPO) in the sciatic nerve and spinal cord.

Sciatic nerve				
Group	GSH (µmol/mg of protein)	GST (µmol CDNB conjugate/min/mg of protein)	iNOS (µmol/mg of protein)	LPO (nmol/TBARS/mg of protein)
ND (10 ml/kg)	48.22 ± 2.1	43.88 ± 1.5	34.22 ± 3.1	62.43 ± 1.8
DM (65 mg/kg)	22.32 ± 1.7##	28.42 ± 2.0#	65.32 ± 3.2##	112.81 ± 2.6###
BBM (15 mg/kg) + DM	40.29 ± 2.2**	36.10 ± 3.4**	55.21 ± 1.6*	92.36 ± 2.8*
BRG (100 mg/kg) + DM	42.34 ± 2.2**	38.24 ± 1.0**	41.04 ± 3.0**	85.16 ± 2.5**
CAR (20 ml/kg) + DM	46.44 ± 3.6**	40.11 ± 3.6**	48.97 ± 1.2**	84.22 ± 1.6**
PBN (30 mg/kg) + DM	42.17 ± 1.2**	38.30 ± 1.4**	53.21 ± 1.0*	88.42 ± 1.4*
**Spinal cord**				
**Group**	**GSH (µmol/mg of protein)**	**GST (µmol CDNB conjugate/min/mg of protein)**	**iNOS (µmol/mg of protein)**	**LPO (nmol/TBARS/mg of protein)**
ND (10 ml/kg)	43.22 ± 1.8	35.71 ± 2.1	41.35 ± 1.2	62.33 ± 1.3
DM (65 mg/kg)	25.12 ± 2.1##	22.81 ± 2.8##	75.11 ± 2.1###	105.18 ± 3.1##
BBM (15 mg/kg) + DM	38.15 ± 2.8**	33.94 ± 1.2**	48.42 ± 3.5***	80.52 ± 1.8***
BRG (100 mg/kg) + DM	39.16 ± 1.6**	34.22 ± 4.2**	63.31 ± 1.8*	87.42 ± 2.6**
CAR (20 ml/kg) + DM	35.26 ± 2.6**	30.55 ± 1.2*	54.81 ± 2.6**	77.54 ± 3.2**
PBN (30 mg/kg) + DM	35.11 ± 2.5**	32.41 ± 1.8*	45.15 ± 2.2***	85.41 ± 2.1**

The data were expressed as mean ± SEM (*n* = 6). One-way ANOVA, followed by *post hoc* Tukey test. ^###^ *p* < 0.001 vs. sham ^*^ *p* < 0.05, ^**^ *p* < 0.01 vs. STZ-induced diabetic neuropathy.

### Histopathological analysis (H&E staining)

**FIGURE 7:**
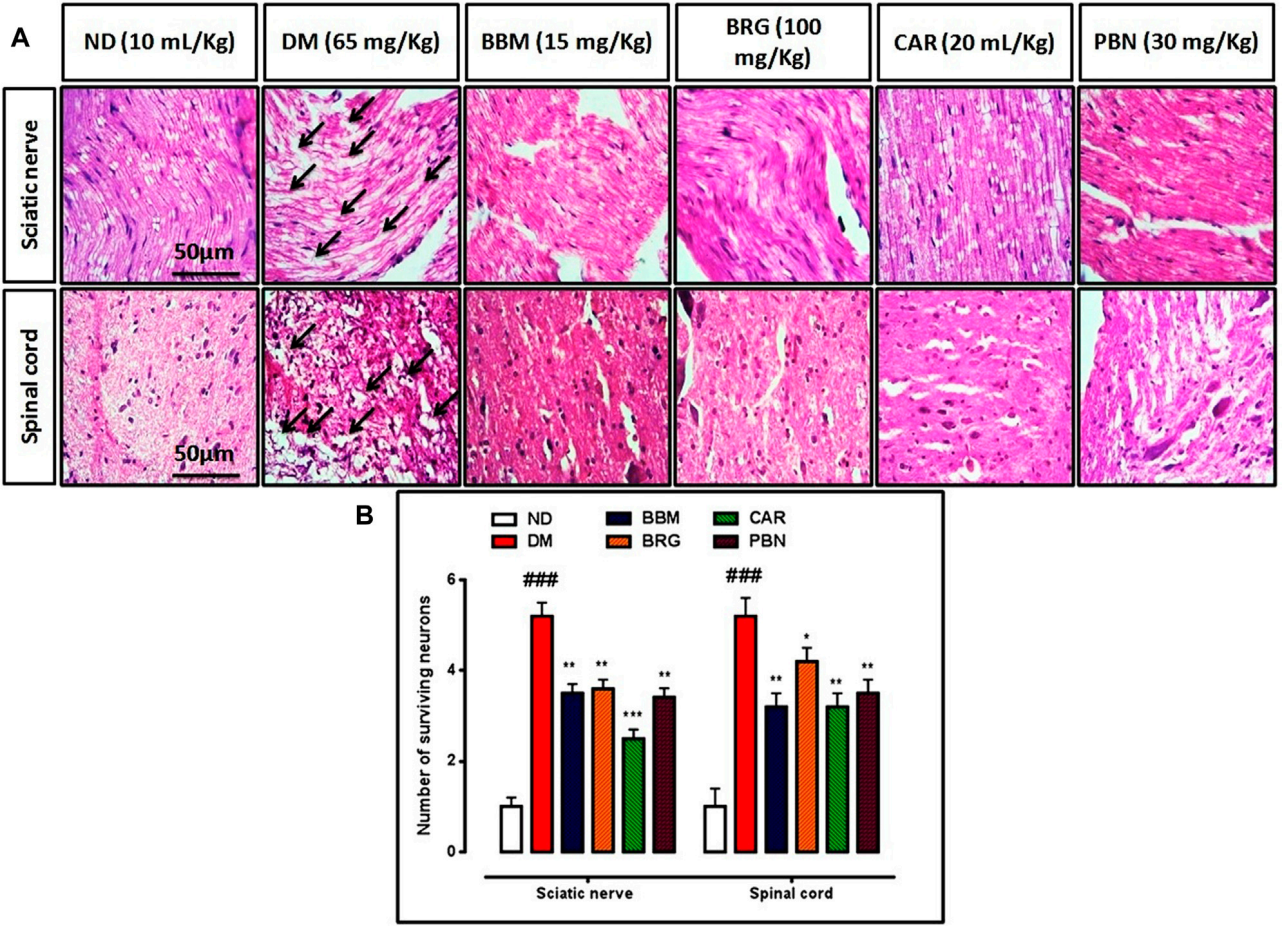
**(A,B)** represents hematoxylin and eosin (H&E) staining of the sciatic nerve and spinal cord and effect of berbamine, bergapten, carveol, and pregabalin on relative integrated density of the sciatic nerve and spinal cord. Images are analyzed by ImageJ software. Bar 50 µm, magnification 40x. GraphPad prism is used to plot the graph and probability value is calculated by Graph pad instate. Data expressed as mean ± SEM, (*n* = 6). One-way ANOVA followed by *post hoc* Tukey test. ^###^ *p* < 0.001 vs. non-diabetic (ND),^*^ *p* < 0.001, ^**^ *p* < 0.001,^***^ *p* < 0.001 vs. streptozotocin-induced diabetic neuropathy (DN).

The CCI group showed injury to the sciatic nerve and spinal cord, with various types of nerve damage, such as cellular spaces, edema formation, and jumbled cellular pattern because of CCI-induced pathological changes. Treatment with berbamine, bergapten, and carveol significantly improved disorientation of the sciatic nerve and spinal cord. Pregabalin also reversed the damage presented in Figures 7A,B.

### Immuno-histochemistry evaluation

**FIGURE 8:**
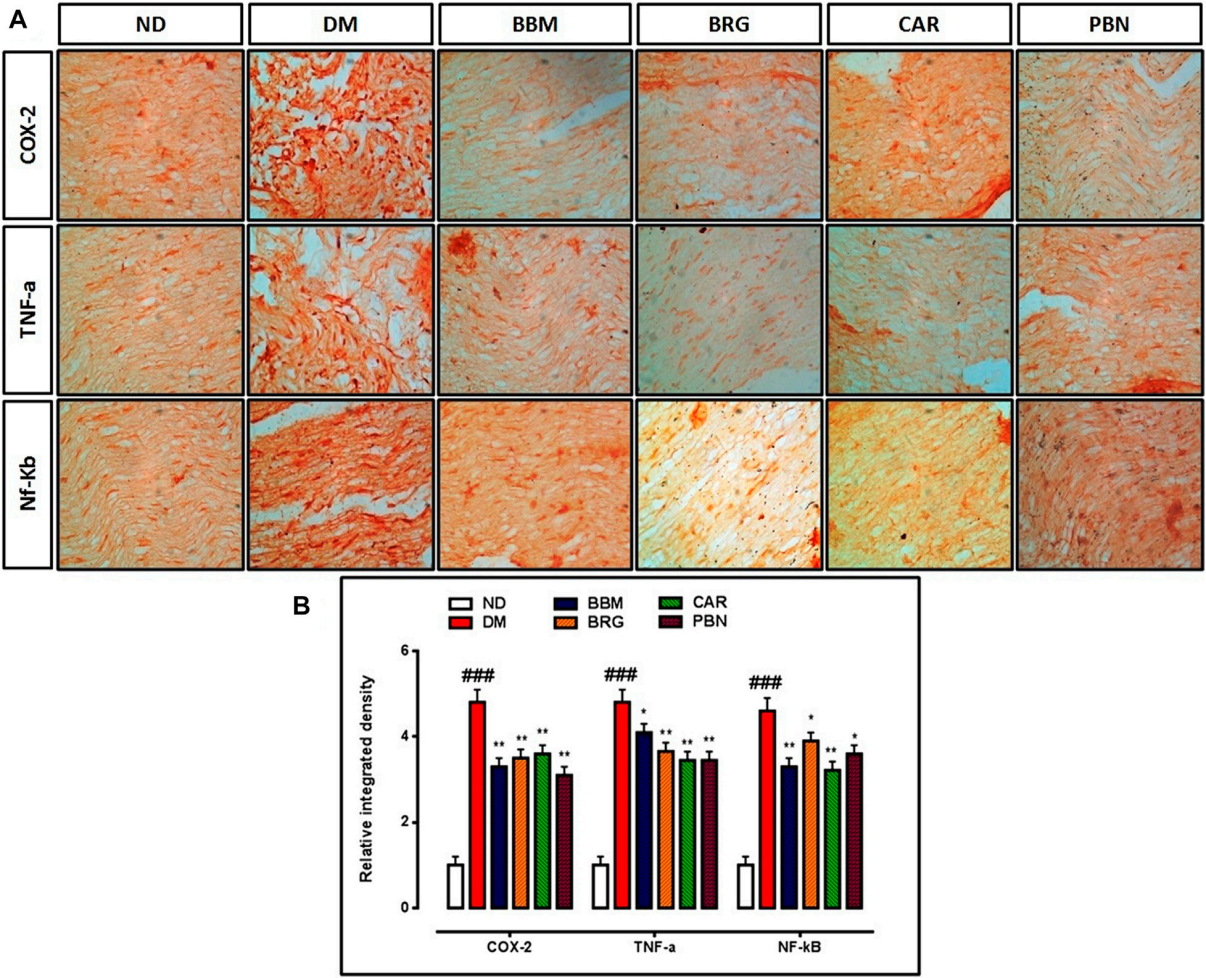
**(A,B)** represent the effect of berbamine, bergapten, carveol, and pregabalin on protein expression of cyclooxygenase-2 (COX-2), tumor necrosis factor-α (TNF-α), and nuclear factor kappa B (NF-κb) in the sciatic nerve. Images were analyzed by ImageJ software Bar 50 µm, magnification 40x. GraphPad prism is used to plot the graph and probability value was calculated by GraphPad instate. Data expressed as mean ± SEM, (*n* = 6). One way ANOVA followed by *post hoc* Tukey test. ^###^ *p* < 0.001 vs. non-diabetic (ND), ^*^ *p* < 0.05 and ^**^ *p* < 0.01 vs. streptozotocin-induced diabetic neuropathy (DN).

Results demonstrate that in collected tissue of the spinal cord there is marked elevation of the COX-2, TNF-α, and NF-κb in the STZ-induced diabetic group vs. saline group. Berbamine, bergapten and carveol attenuated over-expressed levels of COX-2, TNFα, and NF-κb significantly, as shown in Figures 8A,B. The standard drug (PBN) downregulated COX-2, TNF-α, and NF-κb in the spinal cord.

### Effects on inflammatory markers (ELISA)

**FIGURE 9:**
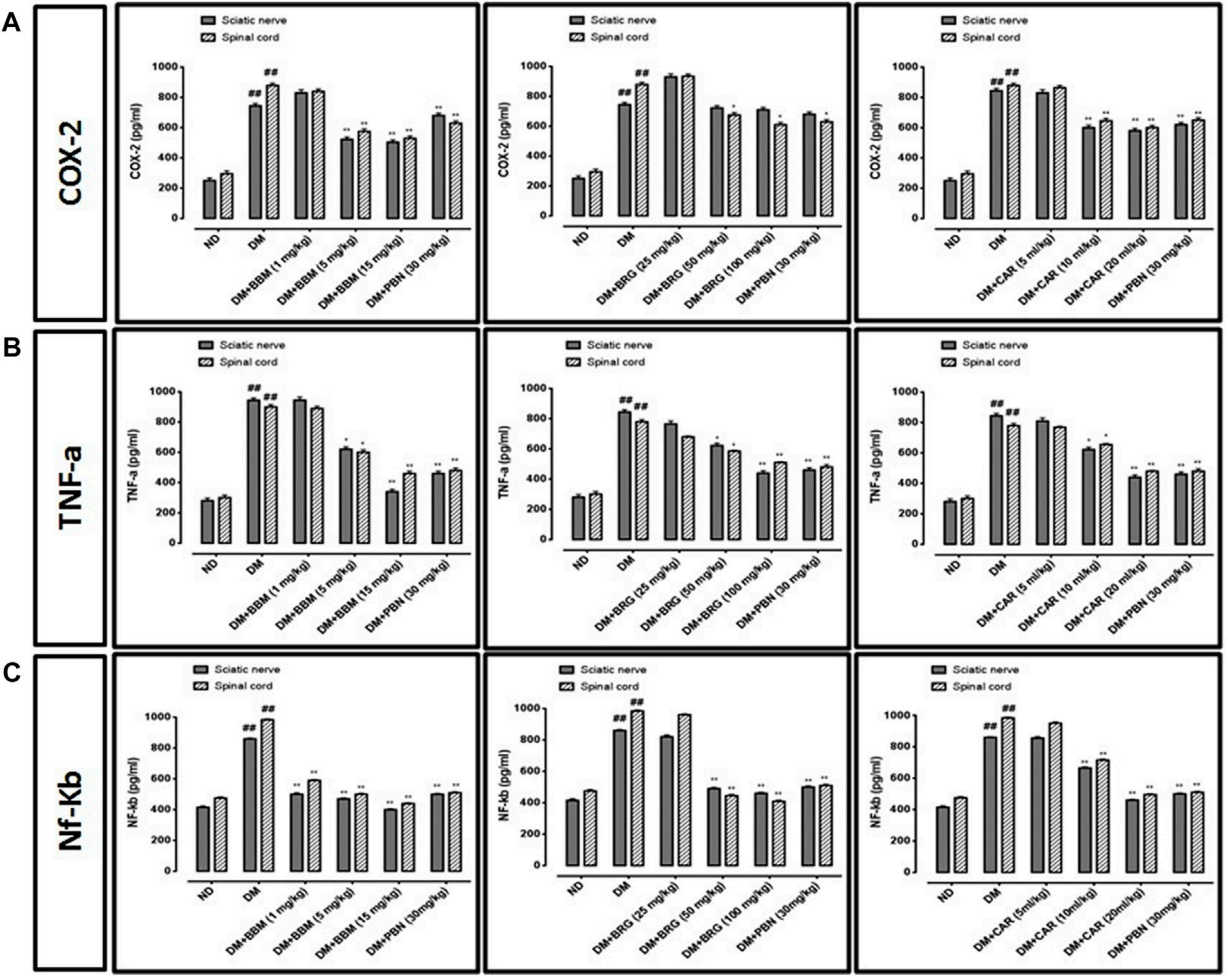
Represents the effect of berbamine, bergapten, carveol, and pregabalin on protein expression of **(A)** cyclooxygenase-2 (COX-2), **(B)** tumor necrosis factor-α (TNF-α), and **(C)** nuclear factor kappa B (NF-κb) in the sciatic nerve and spinal cord quantified by ELISA. Data expressed as mean ± SEM, (*n* = 6). One-way ANOVA followed by *post hoc* Tukey test. ^###^ *p* < 0.001 vs. non-diabetic (ND), ^*^ *p* < 0.05 and ^**^ *p* < 0.01 vs. streptozotocin-induced diabetic neuropathy (DN).

We investigated the effects of berbamine, bergapten, and carveol on the expression of COX-2, TNF-α, and NF-κb in the sciatic nerve and spinal cord as shown in Figures 9A–C. All three markers were overexpressed in the CCI group vs. the saline group in the sciatic nerve and spinal cord. Berbamine (5 and 15 mg/kg), bergapten (50 and 100 mg/kg), and carveol (10 and 20 ml/kg), decreased expression of COX-2 (*p* < 0.01) in the sciatic nerve and spinal cord. Berbamine (5 and 15 mg/kg), bergapten (50 and 100 mg/kg), and carveol (10 and 20 ml/kg), down regulated TNF-α in the sciatic nerve and spinal cord. Berbamine (5 and 15 mg/kg), bergapten (50 and 100 mg/kg), and carveol (10 and 20 ml/kg) lowered the expression of NF-κb in the sciatic nerve and spinal cord. Pregabalin at 30 mg/kg minimized overexpression of all the inflammatory markers, including COX-2, TNF-α, and NF-κb in the sciatic nerve and spinal cord.

### Effect on mRNA expression of TNF-α

**FIGURE 10:**
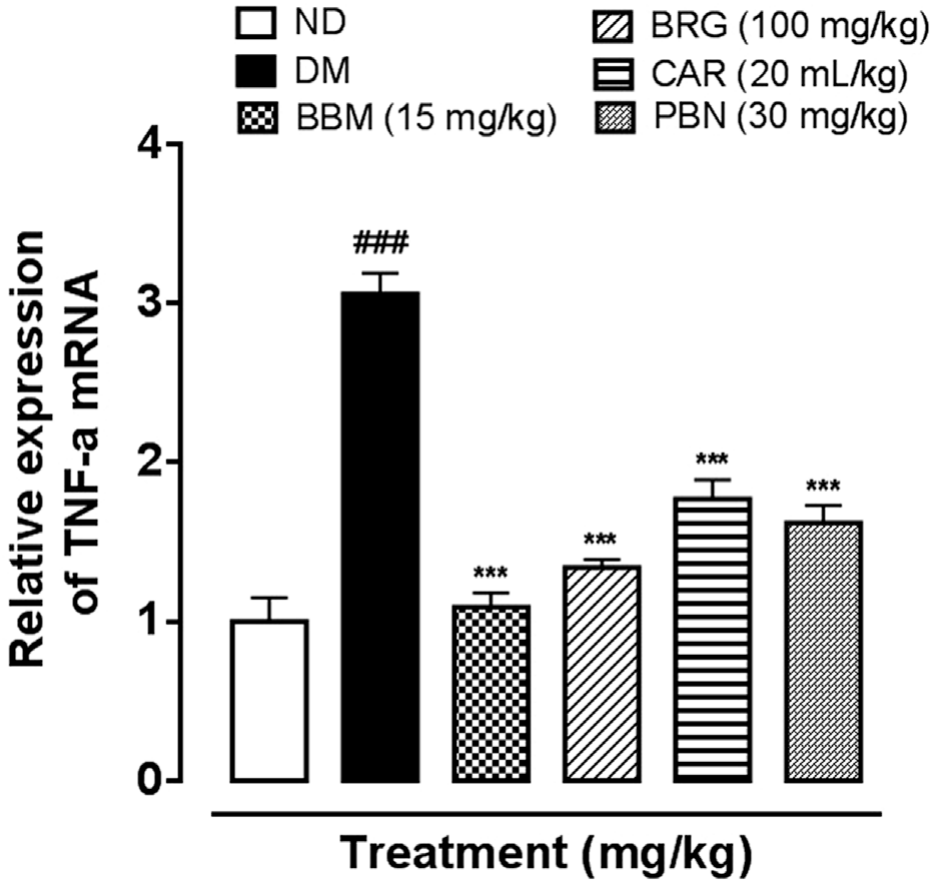
Represents the effect of berbamine, bergapten, carveol, and pregabalin on protein expression of mRNA of tumor necrosis factor-α (TNF-α) in the spinal cord quantified by polymerase chain reaction. Data expressed as mean ± SEM, (*n* = 6). One-way ANOVA followed by *post hoc* Tukey test. ^###^ *p* < 0.001 vs. non-diabetic (ND), ^*^ *p* < 0.05 and ^**^ *p* < 0.01 vs. streptozotocin-induced diabetic neuropathy (DN).

We investigated the effect of BBM, BRG, and CAR on the mRNA expression of TNF-α in the spinal cord as shown in Figure 10. The result revealed that mRNA expression of TNF-α is significantly raised in the DN group. The treatment (BBM, BRG, and CAR) down regulated the mRNA expression of TNF-α as displayed. Pregabalin was used as standard, and found to decrease the mRNA expression of TNF-α.

## Discussion

The STZ-induced diabetic model not only summarized the elevated PGLs and reduced body weight, but rats were also affected by neuropathic pain, making it a perfect model to examine DN. Diabetic neuropathy is one of most widely recognized complications of DM and has multi-factorial causes, and so its pathogenesis is debatable. Consequently, it is a serious challenge for scientists to explore the mechanisms of DN in addition to investigating and assessing new therapeutic alternatives. Nevertheless, oxidative stress and neuroinflammation have been identified as the leading pathophysiological cause in the development of DN (Kumar and Mittal, 2017).

In this study, a thermal-hyperalgesia test was used to confirm DM-induced DN in rats. The DN group had diminished body weight, significantly higher PGLs, and the nociceptive-threshold of thermal hyperalgesia and mechanical allodynia were significantly lower compared to the saline-group, indicating the development of DN. The current research explored neuro-protective effects in DN-induced neuro-inflammation through natural isolates. The outcomes of 14-days of therapy disclose that the behavioral deficit and raised expression of oxidative stress were significantly reversed, and the higher-expressions of inflammatory mediators, such as COX-2 and TNF-α were minimized. To explore the impact of berbamine, bergapten, and carveol on DN, various methods such as *in silico* studies, behavioral investigation, and molecular analysis were used.

In the *in silico* studies, BBM, BRG, and CAR were docked against target MAPKs. The DSV-2016 was used to attain molecular interactions by visualizing the docked ligand and target proteins. The ligand-target complex was significantly stabilized by molecular interaction in the form of hydrogen bonds and hydrophobic bonds (Al Kury et al., 2019). The interactions of BBM, BRG, and CAR constituted hydrogen bonding with binding affinity of 6.9 (BBM), 6.5 (BRG) and 5.8 (CAR) Kcal/mol against the MAPKs. The docking was further validated by utilizing a more advanced technique of molecular dynamic simulation and the results are best explained in Figures 3, 4, 5 and 6. The simulation study also explored how, in a physiological environment, the ligands interact with the target as reflected by their hydrogen bonds. Hence the docking studies are further validated with the simulation, with the compounds exhibiting hydrogen bonding with the same targets (ASP168).

Published research discloses that diminished sensory and motor nerve conduction velocities initiate different signs and symptoms related to peripheral DN, which include numbness induced by chronic hyperglycemia (Edwards et al., 2008). Oxidative stress is the principal pathophysiology associated with the worsening progression of many diseases (Chanchal et al., 2016). In DM, oxidative stress is recognized as a main risk factor for nerve injury, which results in the aggravation of pain impulses that characterize DN (Sandireddy et al., 2014). Diminished antioxidant capability and high oxygen utilization decrease nerve tissues prone to oxidative attack (Singh et al., 2014). It is well reported that oxidative stress in DM causes nerve conduction loss due to loss of myelination, and enormous myelinated fibers cause axonal damage, which decrease the nerve action potential, changing pain discernment, which can be estimated through behavioral investigation, such as thermal-hyperalgesia and mechanical allodynia (Said, 2007). Diabetic neuropathy in the rat model was reduced by minimizing oxidative stress to nerve tissue.

In the STZ-induced DN group, the levels of GSH and GST were significantly decreased and an enormous rise in iNOS and LPO was seen in the sciatic nerve and spinal cord. The sciatic nerve injury resulted from inflammation via the creation of a system of reactive oxygen species (ROS) and elevation of the level of inflammatory mediators. The spinal cord was also damaged due to direct association with the sciatic nerve (Shah et al., 2018). As an important antioxidant, GSH is also a significant controller of intra-cellular redox potential (Musaogullari et al., 2020). The values of GSH were considerably reduced within the sciatic nerves of the STZ-induced DN group (Kishore et al., 2017), but were restored through treatment with BBM, BRG, and CAR. Consequently, the deficit in antioxidant capability in the STZ-induced DN group stimulated free radicals to assault cell membranes, initiate lipid peroxidation, and augment the level of inflammatory nitric oxide, thus damaging the tissue as a consequence of neuropathy (Hosseini and Abdollahi, 2013). The lipid peroxidation augmented by free radicals, which may be generated through glucose-auto-oxidation and protein-glycation (Metz et al., 2003), results in disruption of the antioxidant defense system (Faheem et al., 2021). The development of LPO and iNOS, which is especially associated with diminishing defensive endogenous antioxidants of the body and increasing ROS production, in turn induced inflammation in the spinal cord (Hains and Waxman, 2006). The levels of antioxidants (GSH and GST) were significantly reduced, whereas levels of LPO and iNOS were elevated in the STZ-induced DN group as compared to the saline group. Administration of different doses of BBM, BRG, and CAR ameliorated lipid peroxidation and improved antioxidant status in the sciatic nerve and spinal cord of DN rats *via* increasing levels of antioxidant enzymes.

The principle reason for painful DN is neuro-inflammation, which diminishes the peripheral nerves both structurally and functionally (Calvert et al., 2009). Multiple lines of proof acquired from experimentation and medical studies confirm that neuroinflammation is one of the major reasons for several deficiencies found in DN. In such manner, it was properly reported that peripheral neurons in DM activate *p*- NF-κB that mediates the improved inflammatory response (Wang et al., 2008). Many reviews have proven that in rat tissues of DM, hyperglycemia results in aggregation of advanced glycation end products, which bind with a cellular-receptor and stimulate the progression of *p*-NF-κB. This in turn triggers the production of pro-inflammatory cytokines (TNF-α and COX-2) and the elevation of oxidative stress markers (Schmidt et al., 1999).

Similarly, elevated levels of TNF-α were also observed within the sciatic nerves and spinal cords of the STZ-induced DN group, and suppression of these proinflammatory cytokine levels was accompanied by mitigation of neuropathic pain (Leng et al., 2020). COX-2 has been shown to be involved in the initiation and maintenance of DN (Kellogg et al., 2008). Several examinations have confirmed that TNF-α is an important pro-inflammatory cytokine in the STZ-induced DN group, and might stimulate the nuclear transcription factor NF-κB, which in turn initiates the cellular viability deterioration and stimulates demyelination of nerves (Evangelista et al., 2018). The NF-κB significantly stimulates the overexpression of proinflammatory cytokines and results in nerve damage (Sandireddy et al., 2014). Cyclooxygenase 2 (COX-2), and other inflammatory cytokines, have been regulated by direct involvement of the principle nuclear transcription factor, *p*-NF-κB.

The elevated levels of COX-2 are one more marker associated with the arbitration and progression of thermal hyperalgesia and mechanical allodynia (Boccella et al., 2019). Stimulation of COX-2 *via p*-NF-κB prompts changes to the osmolyte levels in nerves and augmented vasoconstrictors, together with thromboxane accumulation (Pop-Busui et al., 2002). The higher levels of COX-2 can result in an inequality of TXA2/PGI2, which may additionally stimulate hypoxia in tissues, and impairment of nerve tissues both structurally and functionally (Kellogg et al., 2007). Previous studies confirm that in nerve tissues of rats, COX-2 was detected to regulate the Na^+^- K^+^ activity and augment the production of vasoconstrictor thromboxane (Pop-Busui et al., 2002). A significant event in the whole series of changes is represented by the stimulation of the *p*- NF-κB pathway and elevated levels of proteins consisting of TNF-α and COX-2. However, the progression of DN is stated by overexpression of inflammatory markers, such as COX-2 and TNF-α. Inhibition of these inflammatory mediators results in significant decrease in severity of pain (Cheng et al., 2010). In treatment groups, immuno-histochemical assessments showed that elevated expression of COX-2 and TNF, and *p*- NF-κB in the STZ-induced DN group, decreased markedly after treatment with BBM, BRG, and CAR. The morphology of the sciatic nerves in DN rats was also severely destroyed.

In this study, we confirmed STZ-induced inflammatory responses with ELISA, analyzing the NF-κb, COX-2, and TNF-α protein levels in the sciatic nerve and spinal cord of the STZ-induced DN group, and our results confirmed that these proinflammatory cytokines were up regulated in response to STZ administration. Hence, to find out the expression of NF-κb, COX-2, and TNF-α in the saline, DN, and treatment groups, tissue samples of the sciatic nerve were processed for ELISA. The overexpression of NF-κb, COX-2, and TNF-α in the STZ-induced DN group were significant in pro-inflammatory cytokines. PG was also studied for its potential in bringing down the higher expression of NF-κb, COX-2, and TNF-α in DN rats, and for inhibiting the development of disease (Figures 8D,E). Importantly, our study showed that DM-induced NF-κb, COX-2, and TNF-α protein expression in the sciatic nerve was significantly suppressed by treatment, indicating a role for BBM, BRG, and CAR in the suppression of neuroinflammation in the STZ-induced diabetic group. Our findings are indeed consistent with known roles of IO in the inhibition of oxidative stress and inflammation.

In the present study, treatment significantly attenuated thermal hyperalgesia, and mechanical allodynia, but had no significant effect on PGLs in comparison to the DN group. Treatment reduced oxidative stress, promoting neurodegeneration by down regulation of *p*-NF-κB, COX-2, and TNF-α expression towards normal levels, showing that BBM, BRG, and CAR can decrease neuroinflammation. Therefore, focusing on oxidative stress and inflammation pathways will possibly be significant therapeutic methods in the future to diminish the prevalence of DN. In order to find out the impact of BBM, BRG, and CAR in DN, several approaches, along with *in silico*, molecular dynamic simulation, behavioral, and molecular techniques were applied. It was demonstrated that behavioral deficits, and biochemical and inflammatory changes in the rat model of STZ-DM-induced DN were significantly ameliorated with BBM, BRG, and CAR.

## Conclusion

The current research explored the therapeutic potential of BBM, BRG, and CAR in STZ-induced DN. The result revealed that the abovementioned compounds halt neuroinflammation and neurodegeneration by down regulating NF-κB, which in turn minimizes overexpressed levels of proinflammatory cytokines, including TNF-α and COX-2. These findings were confirmed by various molecular investigations, such as IHC, ELISA, and PCR. Thus, BBM, BRG, and CAR may offer a new remedial option for management of neuropathic pain, and for prevention of oxidative stress and neuro-inflammation in STZ-mediated DM-induced neuropathic pain.

## Data Availability

The original contributions presented in the study are included in the article/Supplementary Materials. Further inquiries can be directed to the corresponding authors.

## Abbreviations

BBM, berbamine; BRG, bergapten; CAR, carveol; STZ, streptozotocin; GSH, glutathione; GST, glutathione s-transferase; iNOS, inducible nitric oxide synthase; LPO, lipid peroxidase; H&E, hematoxylin and eosin; IHC, immuno-histochemistry; COX-2, cyclooxygenase; TNF-α, tumor necrosis factor-alpha; NF-κb, nuclear factor kappa B; ELISA, enzyme-linked immunosorbent assay; PBS, phosphate buffer saline; H_2_O_2_, hydrogen peroxide; DTNB, 5, 5′-dithio-bis-2-nitrobenzoic acid; CDNB, 2,4-dinitrochlorobenzene; DAB, diaminobenzidine peroxidase; TCA, trichloroacetic acid; REC, research and ethical committee; IP, intraperitoneally; SN, sciatic nerve; SC, spinal cord; NO, nitric oxide; HRP, horseradish peroxidase; PCR, polymerase chain reaction.
